# Renal Function and Atrial Remodeling: Interpreting Voltage‐Mapping Limitations

**DOI:** 10.1111/anec.70144

**Published:** 2025-12-21

**Authors:** Mücahit Aker, Macit Kalçık, Lütfü Bekar

**Affiliations:** ^1^ Department of Cardiology Hitit University Erol Olçok Education and Research Hospital Corum Turkey; ^2^ Department of Cardiology Faculty of Medicine, Hitit University Corum Turkey

To the Editor,

We read with interest the recent article by Deng et al., which explored the association between renal function and left atrial low‐voltage area (LVA) burden in patients with atrial fibrillation (Deng et al. 2025). The authors report increased LVA prevalence among individuals with reduced estimated glomerular filtration rate (eGFR), suggesting a link between renal dysfunction and atrial substrate remodeling. Although the research addresses an important clinical question, several methodological considerations limit the strength of the conclusions.

A primary issue concerns the heterogeneity and intrinsic variability of bipolar voltage mapping. Voltage measurements are highly sensitive to catheter contact, mapping density, electrode configuration, and rhythm status during acquisition. The study does not clarify mapping stability parameters or point density across participants, factors known to influence LVA quantification and potentially introduce systematic misclassification (Huang et al. 2022). Without standardized acquisition protocols, observed differences in LVA burden may partly reflect procedural variability rather than true substrate differences.

Another limitation arises from the distribution of renal function within the cohort. Only a small minority of subjects appear to have eGFR values in ranges typically associated with structural atrial remodeling. Prior studies have demonstrated that meaningful electrophysiologic atrial alterations are most evident in moderate‐to‐severe renal impairment, rather than in mild reductions of eGFR (Lee et al. 2021). The limited representation of lower renal‐function strata raises concerns regarding statistical power and the generalizability of the renal–atrial substrate association.

The multivariable models also may not sufficiently account for confounders that influence atrial fibrosis and LVA burden. Duration of atrial fibrillation, left atrial volume, hypertension severity, glycemic status, and systemic inflammatory markers are independently associated with voltage reduction and may overlap mechanistically with renal dysfunction. Omitting several of these parameters risks attributing substrate differences to renal function while partially reflecting unmeasured cardiac and metabolic remodeling pathways (Karakasis et al. 2024).

Finally, the causal interpretability of the findings warrants caution. Atrial LVA represents a complex composite of fibrosis, anisotropy, and conduction heterogeneity, whereas renal dysfunction reflects systemic microvascular, neurohormonal, and inflammatory alterations. Although these processes may coexist, current evidence supports correlation rather than directional causality. The study would benefit from integration of imaging‐based fibrosis quantification or biomarkers of extracellular matrix turnover to substantiate mechanistic claims (Pegoraro et al. 2025). As it stands, the prognostic implications of renal function for substrate‐guided ablation strategies remain uncertain.

## Author Contributions

All of the authors contributed planning, writing, and revision.

## Funding

The authors have nothing to report.

## Conflicts of Interest

The authors declare no conflicts of interest.

## Data Availability

Data sharing is not applicable to this article as no datasets were generated or analyzed during the current study.
